# Does antibiotic treatment duration affect the outcomes of exacerbations of asthma and COPD? A systematic review

**DOI:** 10.1177/1479972317745734

**Published:** 2017-12-12

**Authors:** Marie Stolbrink, Jack Amiry, John D Blakey

**Affiliations:** 1 Institute of Infection and Global Health, University of Liverpool, Liverpool, UK; 2 Aintree University Hospital, Liverpool, UK; 3 Department of Respiratory Medicine, Royal Liverpool University Hospital, Liverpool, UK; 4 Health Services Research Institute, Institute of Psychology Health and Society, University of Liverpool, Liverpool, UK

**Keywords:** Asthma, COPD, exacerbation, antibiotics, duration, antimicrobial resistance

## Abstract

Asthma and chronic obstructive pulmonary disease (COPD) cause significant morbidity and mortality worldwide, primarily through exacerbations. Exacerbations are often treated with antibiotics but their optimal course duration is uncertain. Reducing antibiotic duration may influence antimicrobial resistance but risks treatment failure. The objective of this article is to review published literature to investigate whether shorter antibiotic therapy duration affects clinical outcomes in the treatment of asthma and COPD exacerbations. We systematically searched electronic databases (MEDLINE, EMBASE, CINAHL, World Health Organisation International Clinical Trial Registry Platform, the Cochrane library, and ISRCTN) with no language, location, or time restrictions. We retrieved observational and controlled trials comparing different durations of the same oral antibiotic therapy in the treatment of acute exacerbations of asthma or COPD in adults. We found no applicable studies for asthma exacerbations. We included 10 randomized, placebo-controlled trials for COPD patients, all from high-income countries. The commonest studied antibiotic class was fluoroquinolones. Antibiotic courses shorter than 6 days were associated with significantly fewer overall adverse events (risk ratio (RR): 0.84, 95% confidence interval (CI): 0.75–0.93, *p* = 0.001) when compared with those of 7 or more days. There was no statistically significant difference for clinical success or bacteriological eradication in sputum (RR: 1.00, 95% CI: 0.88–1.13 and RR: 1.06, 95% CI: 0.79–1.44, respectively). Shorter durations of antibiotics for COPD exacerbations do not seem to confer a higher risk of treatment failure but are associated with fewer adverse events. This is in keeping with previous studies in community acquired pneumonia, but studies were heterogeneous and differed from usual clinical practice. Further observational and prospective work is needed to explore the significance of antibiotic duration in the treatment of asthma and COPD exacerbations.

## Introduction

### Background

Asthma and chronic obstructive pulmonary disease (COPD) are common and are becoming more prevalent globally.^1–4^ Exacerbations are a major driver of the morbidity, mortality, and cost associated with these chronic airways diseases.^5,6^ The majority of exacerbations are nonbacterial in origin.^7–9^ They are, however, frequently treated with antibiotics hence causing a significant antibiotic burden.^10,11^ For example, over 11 years, 22% of 16.1 million asthma presentations to US hospitals received antibiotics, largely against current guidelines.^12^

Antimicrobial resistance is one of the most important public health crises facing the world today. Reduced susceptibility to penicillin or penicillin-resistance in *Streptococcus pneumoniae* exceeds more than 50% in many countries.^13^ The World Health Organisation (WHO) issued a global action plan on antimicrobial resistance in 2015 which called for optimization of antibiotic prescribing.^14^ Use of shorter antibiotic courses may be beneficial to reduce resistance, improve concordance, costs, and side effects. However, shorter courses risk treatment failure. Patients in middle and lower income countries are more susceptible to failure due to a number of factors: reduced susceptibility to penicillins; limited access to follow-up; malnutrition; and higher risk of abnormal lung architecture caused by air pollution, smoking, and industrial exposures.^15–17^

The ideal duration of antibiotic treatment for asthma and COPD exacerbations is uncertain and a prescribing consensus is a priority for providers. The last systematic review on antibiotic duration in COPD exacerbations was published in 2008^18^ and none have been published in asthma exacerbations. We therefore undertook an up-to-date review and meta-analysis to investigate whether shorter courses of oral antibiotic treatment for asthma and COPD exacerbations are associated with different outcomes when compared with longer courses.

### Methods

#### Data sources and search strategy

We conducted systematic searches of bibliographic databases including MEDLINE, EMBASE, and CINAHL through National Health Service library services. We also ran a search of the WHO International Clinical Trial Registry Platform, the Cochrane library, and ISRCTN and used search engines on their own websites. All databases were searched from inception until February 29, 2016. There was no restriction of publication language. We removed duplicate references using reference management software (EndNote X7; Thomson Reuters, USA). The search strategy is described below. The reference lists of earlier reviews on the same topic and abstracts of the European Respiratory Society and American Thoracic Society conferences from the previous year (2015) were hand-searched and titles included if the inclusion criteria were fulfilled.

#### Study selection

We included observational and controlled trials of adults (≥ 18 years) with a clinical diagnosis of asthma or COPD exacerbation. We only included original studies with explicitly different durations of the same oral antibiotic therapy. We excluded the studies of pneumonia treatment and prophylactic antibiotics.

We reviewed the list of titles to exclude publications which were clearly not contributory on this basis and duplicate titles. Two investigators screened the eligible abstracts independently. We obtained full text articles of selected papers via University of Liverpool library, NHS library, and interlibrary loans. Two investigators reviewed the full texts for eligibility independently. Any disagreement was resolved by a third investigator.

#### Data extraction

Two authors extracted data using a preset data extraction form which included details of the study’s publication, authorship, and funding; study characteristics (design and location); participants (sample size, method of recruitment, selection, and demographics); outcome measures; interventions; data analysis and reporting; confounding adjustments; and the main findings. Disagreements were resolved by discussion. We used RevMan 5.3 (Cochrane Collaboration) and EndNote X7 software in the collection and management of data from abstracts and papers.

#### Quality and risk of bias assessment

We assessed the studies’ accuracy and risk of bias using the Cochrane Handbook for Systematic Reviews of Interventions criteria.^19^ Controlled trials were additionally analyzed using the Cochrane Collaboration’s tool for assessing risk of bias.

#### Analysis

We combined intention to treat population data from comparable studies in quantitative analyses. We pooled data using fixed effect model in RevMan 5.3.^20^ We used the Mantel–Haenszel method, presenting data as risk ratio (RR) with 95% confidence intervals (CIs). We used statistical significance of *p* < 0.05 and assessed the degree of heterogeneity using the I^2^ statistic.

## Results

### Asthma

**Figure 1.**
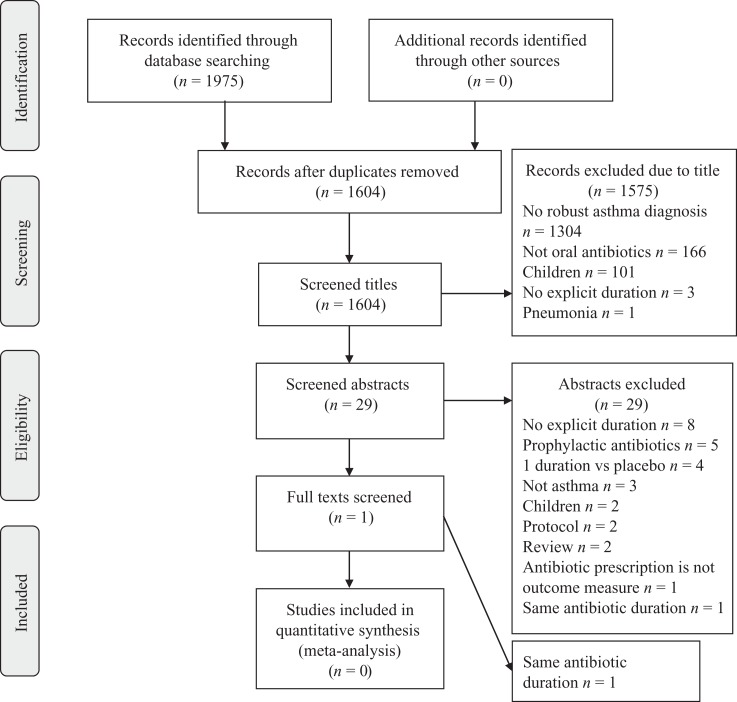
PRISMA flow diagram of systematic search for asthma studies.

We identified 1604 individual titles through database searches (Figure 1). No additional studies were identified by hand-searching. The commonest reason for noninclusion into abstract screening was a lack of an asthma diagnosis for all participants (1304 records). We reviewed 29 abstracts. The commonest reason for not progression to full text review was missing explicit antibiotic duration (eight studies), with other reasons demonstrated in Figure 1. The one full text analyzed assessed antibiotics for one duration only and was hence excluded.

### COPD

**Figure 2.**
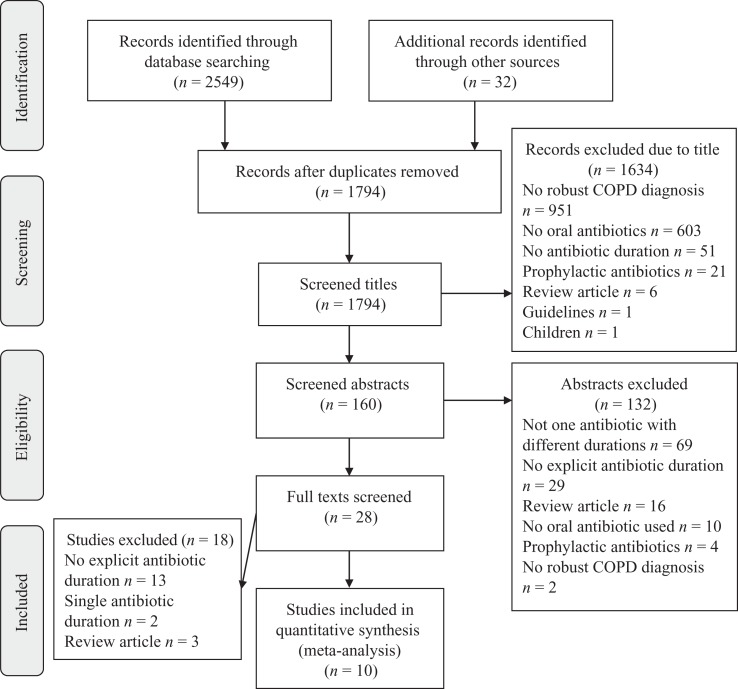
PRISMA flow diagram of systematic search for COPD studies. COPD: chronic obstructive pulmonary disease.

We identified 1762 individual titles in COPD from database searches, 32 from hand-searching of recent reviews and 2 from conference abstracts (Figure 2).^18,21^ The commonest reason for noninclusion in abstract screening was lack of COPD diagnosis (951 studies) and not assessing oral antibiotic treatment (603 studies). We screened 160 abstracts. The commonest reasons for exclusion at this stage were not assessing one antibiotic with two different durations (67 studies) or not comparing specific antibiotic durations (26 studies). Thirty-three full texts were eligible for analysis and 10 full texts were included in the final meta-analysis. One text in Polish was translated but was not applicable.

### Characteristics and definitions of COPD studies

#### Design, participants, and setting

All 10 studies included in the meta-analysis were randomized, placebo-controlled trials (Table 1). Nine studies considered “chronic bronchitis” but included individuals with airflow limitation and a smoking history: their design would have predated the global use of the term “COPD.”^32^ Eight studies reported smoking status. The youngest enrolled participant was 18 years old. Four studies recruited from outpatients, three from hospital admissions, one from primary care, and two from primary care and outpatients. Two multicenter studies included patients in the developing world (Latin America, Pakistan, Philippines),^29,31^ and the rest were based in Europe or North America. Where documented, all exacerbations were diagnosed clinically, one study used microscopically confirmed purulent sputum.^26^ There was a range of exacerbation severities from “mild” in outpatients to inpatients not needing critical care or ventilation.^26,30^ Eight studies characterized the severity of the underlying lung disease.

**Table 1.** Characteristics of included full text studies.

Study design	Study name	Location	Sample selection and setting	Sample size	Antibiotic and durations	Year	Recruitment	Outcome measures
RCT	Bennett et al.^22^	United Kingdom (1 center)	Disease: chronic bronchitis Diagnosis made: **“**as defined by Medical Research Council (1965)” Exacerbation diagnosis: not specified—likely clinical Exacerbation severity: not specified Randomization: unclear LTFU: 5% Smokers; age: “all past or current smokers”; youngest patient 40 years	41	Amoxicillin 3 g twice daily for 3 days versus 500 mg three times daily for 7 days	January 1984–March 1985	Admissions to one hospital	Duration of hospital admission Reduction in sputum volume, pus or mucous Change in forced expiratory volume in 1 second (FEV1) Treatment failure Time to first exacerbation Mean number of exacerbations Number of deaths over 1 year Number of deaths during admission
RCT	Chodosh et al.^23^	USA (56 centers)	Disease: chronic bronchitis and COPD Diagnosis made**:** clinically for chronic bronchitis; unspecified for COPD Exacerbation diagnosis: clinical Exacerbation severity: mild-moderate (I–III American Thoracic Society and Anthonisen category) Randomization: Simple random allocation LTFU: 9% Smokers; age: **“**yes” (86% short course, 83% long course); youngest patient 19 years	614	Moxifloxacin 400 mg once daily for 5 days versus 7 days	November 1996–April 1998	Outpatients	Clinical response Sputum microbiology Adverse effects
RCT	Graham et al.^24^	USA (29 centers)	Disease: chronic bronchitis Diagnosis made: clinically Exacerbation diagnosis: clinical Exacerbation severity: not specified Randomization: simple random allocation LTFU: 22% Smokers; age: not specified; youngest patient 19.4 years	389	Grepafloxacin 400 mg once daily for 5 days versus 7 days	Not given	Outpatients	Clinical response Sputum microbiology Adverse effects
RCT	Johnston et al.^25^	USA (35 sites)	Disease: chronic bronchitis Diagnosis made: clinically Exacerbation diagnosis: clinically Exacerbation severity: nonhospitalized patients only; Anthonisen I–III Randomization: stratified random allocation LTFU: 13% Smokers; age: 68% short course, 63% long course current smokers; youngest patient 18 years	349	Gatifloxacin 400 mg once daily for 5 days versus 7 days (secondary outcome)	November 1998–July 1999	Primary care	Clinical response Adverse effects (nausea and diarrhoea only) Sputum microbiology
RCT	Gotfried et al.^26^	North America (96 sites)	Disease: chronic bronchitis and COPD Diagnosis made: clinically for chronic bronchitis; radiography and spirometry for COPD Exacerbation diagnosis: clinically and microscopically confirmed purulent sputum Exacerbation severity: mild (Anthonisen I and II criteria) Randomization: simple random allocation at each site LTFU: 12% Smokers; age: 89% short course, 88% long course current or previous smoker; youngest patient 18 years	444	Clarithromycin extended-release 1 g once daily for 5 days versus immediate-release 500 mg twice daily for 7 days	December 2002–April 2004	“Ambulatory patients”—presumed outpatients	Clinical cure Sputum microbiology Adverse events Recurrence or superinfection
RCT	Langan et al.^27^	Belgium, Canada, Czech Republic, France, Germany, Poland, Portugal, Spain, United Kingdom (78 centers)	Diagnosis: chronic bronchitis Diagnosis made: presumed clinical Exacerbation diagnosis: clinically Exacerbation severity: nonhospitalized patients only Randomization: not specified—”randomised” LTFU: 27% Smokers; age: not specified; youngest patient 19 years	541	Grepafloxacin 400 mg once daily for 5 days versus 10 days	Not given	Outpatients	Clinical response Sputum microbiology Adverse effects
RCT	Lorenz et al.^28^	Germany (“multicenter”)	Diagnosis: chronic bronchitis Diagnosis made: not specified Exacerbation diagnosis: clinically Exacerbation severity: “highest grade of Anthonisen exacerbation” (type I) Randomization: not specified—”randomised” LTFU: 23% Smokers; age: 25% short course, 24% long course current smoker; youngest patient not specified	217	Cefixime 400 mg once daily for 5 days versus 10 days	Not given	Not documented— presumed hospital	Clinical response Sputum microbiology Change in spirometry and FEV1 Inflammatory markers Adverse effects
RCT	Masterton and Burley^29^	10 countries (seven in Europe, three in Latin America; 48 centers)	Diagnosis: chronic bronchitis Diagnosis made: clinically Exacerbation diagnosis: clinically Exacerbation severity: clinically, mild-severe Randomization: unclear—”random” LTFU: 9% Smokers; age: 63% short course, 64% long course current or ex-smokers; youngest patient 18 years	530	Levofloxacin 500 mg once daily for 5 days versus 7 days	Not given	Primary care or outpatients	Clinical response Sputum microbiology Adverse effects
RCT	Roede et al.^30^	Netherlands (6 centers)	Diagnosis: COPD Diagnosis made: clinically Exacerbation diagnosis: clinically Exacerbation severity: all not needing ventilation or critical care Randomization: Cluster random sample selection LTFU: 19% at day 21 44% at 3 months Smokers; age: 48% short course, 60% long course smokers; youngest patient not specified	48	Co-amoxiclav 625 mg for 3 days versus 10 days (NB: first 3 days could have been intravenous antibiotics)	November 2000– December 2003	“Hospital”—not specified	Clinical response Sputum microbiology Adverse effects Repeat antibiotic prescription Symptom scores Oxygen use Use of concomitant medications
RCT	Sethi et al.^31^	85 centers in Belgium, Canada, Czech Republic, France, Germany, Hong Kong, Pakistan, Philippines, Poland, Romania, Singapore, Switzerland, Taiwan, and USA	Diagnosis: chronic bronchitis Diagnosis made: clinically, GOLD criteria for severity Exacerbation diagnosis: clinically Exacerbation severity: not specified Randomization: unclear—“randomised” LTFU: 8% Smokers; age: 76.7% short course, 76.7% long course ever smoker; youngest patient 32 years	893	Co-amoxiclav 2000/125 mg twice daily for 5 days versus co-amoxiclav 875/125 mg twice daily for 7 days	November 2001–May 2002	“Community” and “hospital”—not specified	Clinical success Sputum microbiology Adverse events

RCT: randomized controlled trial; COPD: chronic obstructive pulmonary disease; LTFU: lost to follow-up.

#### Interventions and outcomes

Fluoroquinolones were the most commonly examined antimicrobial class (five studies). Two studies assessed grepafloxacin and two assessed co-amoxiclav. The shortest antibiotic treatment was 3 days, the longest 10 days. One study included the potential administration of intravenous antibiotic in the first 3 days of treatment.^30^ Follow-up duration varied from 0 days to one year to 3 days after final treatment for 1 year. All studies reported clinical responses (based on sputum production and appearance) and adverse effects. Nine studies assessed changes in sputum microbiology. Spirometry and inflammatory markers were assessed by a smaller number of studies. No studies compared outcomes in high versus low or middle income countries.

#### Risk of bias

One study had low risk of bias across all domains (Table 2).^26^ One study had a high risk of bias due to not considering smoking as a confounding factor.^27^ Two studies did not recruit enough patients for the primary end point based on their power calculations.^30,22^

**Table 2.** Table of risk of bias and study analysis methods.

Study	Blinding/analysis method	Adjustment/confounders	Multiple testing	Risk of bias		Cochrane Collaboration’s tool for assessing risk of bias	
Bennett et al.^22^	Blinding: unclear—“double blind” but all examined by the same physician Analysis per risk factor	Poorly addressed but “all smoking or ex-smoker”	Few analyses	Funding	Commercial	Random sequence generation	Unclear
				Selection	Unclear	Allocation concealment	Unclear
				Response	Low	Blinding of participants and personnel	Unclear
				Follow-up	Unclear	Blinding of outcome assessment	Unclear
				Reporting	Low	Attrition bias	Unclear
				Allocation	Unclear	Reporting bias	Low
				Other	High: under-powered study	Other	
Chodosh et al.^23^	Blinding: unclear— “adherence to pre-defined criteria by assessor” Analysis per risk factor	Adequately addressed	Few analyses	Funding	Commercial	Random sequence generation	Low
				Selection	Low	Allocation concealment	Low
				Response	Low	Blinding of participants and personnel	Unclear for personnel, low for participants
				Follow-up	Low	Blinding of outcome assessment	Low
				Reporting	Low	Attrition bias	Low
				Allocation	Low	Reporting bias	Low
Graham et al.^24^	Blinding: unclear for personnel/statistician Analysis per risk factor	Poorly addressed	Few analyses	Funding	Commercial	Random sequence generation	Low
				Selection	Low	Allocation concealment	Low
				Response	Low	Blinding of participants and personnel	Unclear
				Follow-up	Low	Blinding of outcome assessment	Unclear
				Reporting	Low	Attrition bias	Low
				Allocation	Low	Reporting bias	Low
Gotfried et al.^25^	Blinding: unclear for statisticians Analysis per risk factor	Poorly addressed	Few analyses	Funding	Commercial	Random sequence generation	Low
				Selection	Low	Allocation concealment	Low
				Response	Low	Blinding of participants and personnel	Unclear
				Follow-up	Unclear	Blinding of outcome assessment	Unclear for statisticians
				Reporting	Unclear	Attrition bias	Unclear—not specified how many people were lost during study
				Allocation	Low	Reporting bias	Unclear
Johnston et al.^26^	Blinding: good Analysis per risk factor	Adequately addressed	Few analyses	Funding	Commercial	Random sequence generation	Low
				Selection	Low	Allocation concealment	Low
				Response	Low	Blinding of participants and personnel	Low
				Follow-up	Low	Blinding of outcome assessment	Low
				Reporting	Low	Attrition bias	Low
				Allocation	Low	Reporting bias	Low
Langan et al.^27^	Blinding: unclear for personnel Analysis per risk factor	Poorly addressed	Few analyses	Funding	Commercial	Random sequence generation	Unclear
				Selection	Unclear: not specified	Allocation concealment	Unclear
				Response	Low	Blinding of participants and personnel	Unclear
				Follow-up	Unclear: high LTFU	Blinding of outcome assessment	Unclear for personnel
				Reporting	Low	Attrition bias	Unclear for loss to follow-up
				Allocation	High: confounding factor smoking was not considered	Reporting bias	Low
Lorenz et al.^28^	Blinding: unclear for assessors and statisticians Analysis per risk factor	Poorly addressed	Few analyses	Funding	Commercial	Random sequence generation	Unclear
				Selection	Unclear—not specified	Allocation concealment	Unclear
				Response	Low	Blinding of participants and personnel	Unclear for personnel
				Follow-up	Unclear: 23% LTFU	Blinding of outcome assessment	Unclear
				Reporting	High: per protocol population used for most analyses	Attrition bias	Unclear: per protocol analysis for secondary variables
				Allocation	Unclear	Reporting bias	Unclear: per protocol analysis
Masterton and Burley^29^	Blinding: good Analysis per risk factor	Adequately addressed	Few analyses	Funding	Commercial	Random sequence generation	Unclear
				Selection	Low	Allocation concealment	Unclear
				Response	Low	Blinding of participants and personnel	Unclear for personnel
				Follow-up	Low	Blinding of outcome assessment	Unclear
				Reporting	Low	Attrition bias	Low
				Allocation	Unclear	Reporting bias	Low
Roede et al.^30^	Blinding: good Analysis per risk factor	Adequately addressed	Few analyses	Funding	Commercial and noncommercial	Random sequence generation	Low
				Selection	Low	Allocation concealment	Low
				Response	Low	Blinding of participants and personnel	Low
				Follow-up	Low	Blinding of outcome assessment	Unclear
				Reporting	Low	Attrition bias	Low
				Allocation	Low	Reporting bias	Low
				Other	High risk: unable to recruit enough patients to power study		
Sethi et al.^31^	Blinding: unclear for personnel Analysis per risk factor	Adequately addressed	Few analyses	Funding	Commercial	Random sequence generation	Unclear
				Selection	Unclear: population from which recruited not specified	Allocation concealment	Unclear
				Response	Low	Blinding of participants and personnel	Unclear for personnel
				Follow-up	Low	Blinding of outcome assessment	Unclear
				Reporting	Unclear	Attrition bias	Low
				Allocation	Unclear	Reporting bias	Low
				Other	Unclear: clinical outcome of “failure” was assigned to participants who were LTFU or did not consent to clinical examination	Other	Unclear: patients with previous antibiotic use were not excluded

LTFU: lost to follow-up.

Those with unclear risk of bias lacked information on blinding of participants, personnel, and outcome assessments (8 of 10 studies). All studies were commercially funded, one additionally received noncommercial funding.^30^ We did not detect publication bias.

### Analysis

Combining the populations of the 10 included studies, 1990 patients received short antibiotic courses (fewer than 6 days) and 1989 patients received long courses (7 or more days).

#### Clinical response

Nine studies used the resolution of clinical signs or symptoms of acute exacerbations as their primary outcome. Bennett et al.^22^ used the absence of mucoid sputum in isolation and was hence excluded from meta-analysis for this outcome. Some studies reported outcomes at multiple time points so we presented clinical success as early (within 6 days of treatment completion), middle (7–14 days after treatment completion), or late (more than 20 days after treatment completion). Two studies assessed outcomes at 7–17 days and 17–23 days after treatment completion—they were excluded from this analysis.^23,28^

**Figure 3.**
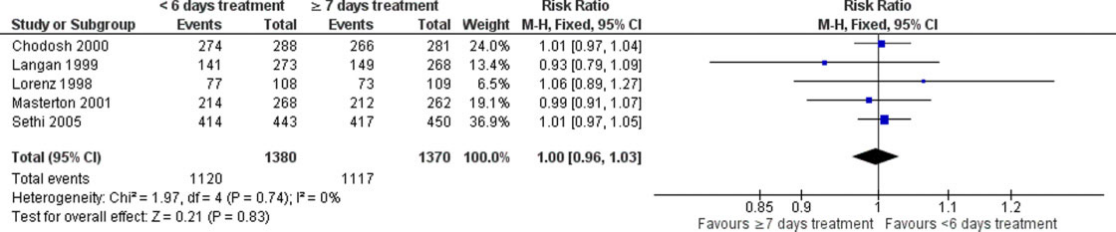
Forest plot of early clinical success, within 6 days of treatment completion, <6 versus ≥7 days antibiotic duration.

There was no statistically significant difference between shorter and longer antibiotic courses in early clinical success (RR: 1.00, 95% CI: 0.96–1.03) in the five studies that considered this (Figure 3).

**Figure 4.**
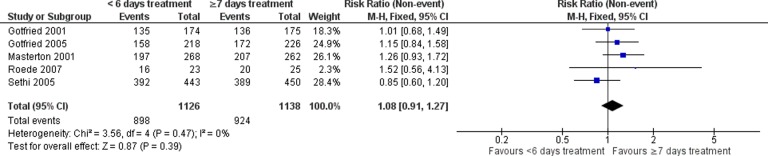
Forest plot of medium clinical success, 7–14 days after treatment completion, <6 versus ≥7 days antibiotic duration.

**Figure 5.**
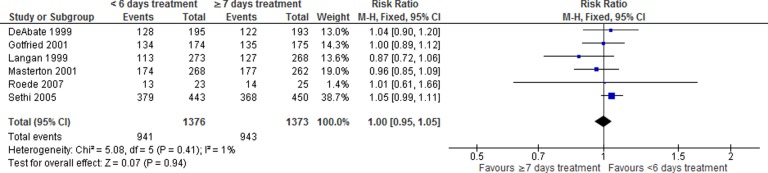
Forest plot of late clinical success, >20 days after treatment completion, <6 versus ≥7 days antibiotic duration.

There was no statistically significant difference in medium (RR: 1.08, 95% CI: 0.91–1.27; five studies; Figure 4) or late clinical success (RR: 1.00, 95% CI: 0.99–1.11; six studies; Figure 5).

#### Adverse events

**Figure 6.**
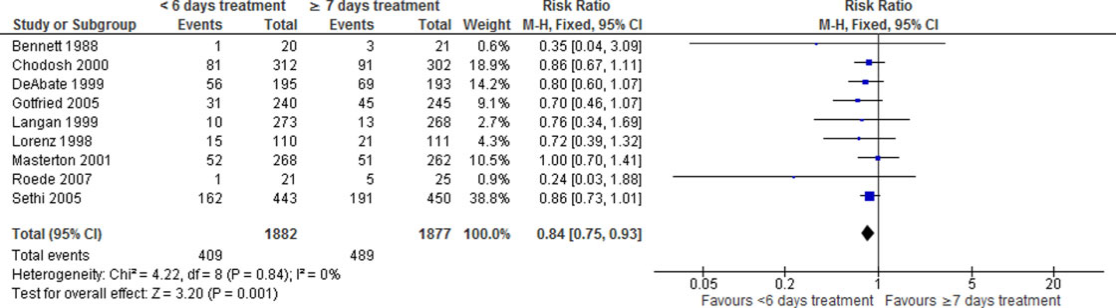
Forest plot of overall adverse events, <6 versus ≥7 days antibiotic duration.

Nine studies reported overall adverse events (1882 and 1877 patients for the shorter and longer duration, respectively). There was a statistically significant lower risk of developing adverse events in the shorter treatment group compared with the longer treatment group (RR: 0.84, 95% CI: 0.75–0.93, *p* = 0.001; Figure 6). For nausea, the risk was statistically significantly lower in the shorter treatment group (RR: 0.71, 95% CI: 0.52–0.98, *p* = 0.04; eight studies; Online Supplemental Material). No significant difference was found for diarrhea (RR: 1.03, 95% CI: 0.82–1.29; seven studies; see Online Supplemental Material).

#### Bacteriological response in sputum

Eight studies assessed eradication or presumed eradication of pathogens which were present in pretreatment sputum samples. Presumed eradication was defined as improvement in clinical symptoms without sputum that could be cultured at follow-up. All studies used populations that had an identified pretreatment pathogen in sputum. There was no statistically significant difference between shorter and longer antibiotic treatment 0–6 days after treatment completion (RR: 1.08, 95% CI: 0.71–1.65; three studies) and 7–23 days after treatment completion (RR: 1.08, 95%: CI 0.83–1.39; seven studies; both in Online Supplemental Material).

#### Other outcomes

Two studies considered spirometric change—there was no statistically significant change in either study between shorter and longer durations.^22,28^ One study assessed inflammatory markers, showing no difference between the different durations.^28^ One study followed patients up to 1 year, assessing occurrence and time to new exacerbations, demonstrating no statistically significant differences between the groups.^22^ Two studies included patients in the developing world but no subgroup analyses for these were reported.^29,31^

## Discussion

### Summary of main findings

The prescription of antibiotics for COPD or asthma exacerbations is a very common clinical activity with serious potential adverse effects. Despite this, we found few studies had investigated optimal antibiotic duration for this indication. There was no difference in clinical success or bacteriological eradication for patients receiving fewer than 6 or more than 7 days of antibiotics for COPD exacerbations. There was, however, a significantly lower risk of side effects overall and specifically nausea in the shorter duration group.

### Strengths and limitations

This review was undertaken systematically following best practice guidance from the Cochrane Collaboration. Interpretation of our findings should be made in the context of the analyzed studies having been largely undertaken at a time of significant variation in the diagnosis and treatment of COPD. The populations were therefore heterogeneous in key aspects such as smoking exposure and airflow obstruction (or did not have these clearly recorded) and by description of exacerbation outcomes. It is plausible that some study participants had other diagnoses such as bronchiectasis and chronic asthma. As many studies were undertaken prior to widespread use of standards for the assessment of COPD, it was not possible to stratify results by severity or GOLD criteria.

A sensitivity analysis without the study where the confounding factor smoking caused a high risk of bias showed no difference in our main findings (see Online Supplemental Material). Most of the older studies also had an “unclear” risk of bias by current standards. Fluoroquinolones were the commonest antibiotic class studied, but these are not first line treatment for uncomplicated exacerbations in usual clinical practice. This diminishes the external validity of the findings.^33^

### Setting in existing literature

Antibiotic courses of 5 or fewer days were as successful as longer courses for clinical and bacteriological cure for chronic bronchitis and COPD exacerbations in a meta-analysis in 2006. However, this study compared course length regardless of drug (e.g., 10 days of cefuroxime with 5 days of telithromycin).^21^ Our study adds to the literature by including a search of the last 10 years of medical publications and by restricting analyses to consider only whether shorter courses of the same antibiotic are as effective and well tolerated. This reduces bias created by different mechanisms of action irrespective of duration.

Shorter courses of antibiotics have already been found to be as effective as longer courses in community acquired pneumonia and pharyngitis, and our findings are consistent with this.^18,21,34–36^

Antibiotics are not routinely recommended for the treatment of asthma exacerbations, and three published studies suggest no benefit above placebo.^7,10,25,37^ However, antibiotics continue to be prescribed extensively for asthma exacerbations.^10,12^ This discrepancy between observed antibiotic prescribing and limited available evidence highlights the need for further studies.

### Implications for future research

This review supports the use of shorter courses of antibiotics for the treatment of COPD exacerbations. However, further research is required to ascertain if these findings hold true in the context of current COPD care, antibiotic use, and antibiotic resistance patterns. The development of extensive electronic health record databases of routinely collected data could be used to provide initial evidence in this regard and could support the design of targeted interventional studies. Future studies in high-income countries are likely to also include biomarker-guided treatment. However, significant challenges exist in lower and middle income countries where antibiotic resistance is prevalent and only fixed duration regimens are currently feasible.

## Conclusions

This systematic review highlights the paucity of research evidence relevant to usual clinical practice informing selection of antibiotic duration for asthma and COPD exacerbations. It appears that courses of antibiotics of 6 or fewer days are equally as effective as those of 1week or longer, but associated with fewer side effects. However, due to the limitations of the published studies, new observational and interventional studies are needed to robustly inform guidelines.

## Supplemental material

Supplemental Material, Review_COPD_Asthma_Abx_supplementary_data_1.0_-_revised_1.0 - Does antibiotic treatment duration affect the outcomes of exacerbations of asthma and COPD? A systematic review Click here for additional data file. Supplemental Material, Review_COPD_Asthma_Abx_supplementary_data_1.0_-_revised_1.0 for Does antibiotic treatment duration affect the outcomes of exacerbations of asthma and COPD? A systematic review by Marie Stolbrink, Jack Amiry and John D Blakey in Chronic Respiratory Disease

## References

[1] Global Asthma Network. Global Asthma Report. http://globalasthmareport.org/ (2014, accessed 20 December 2016).

[2] Institute for Health Metrics and Evaluation. Global Burden of Disease Study. Global Burden of Disease Study. http://ghdx.healthdata.org/gbd-2015 (2015, accessed 21 December 2016).

[3] World Health Organization. Factsheet: Chronic obstructive pulmonary disease (COPD). http://www.who.int/mediacentre/factsheets/fs315/en/ (2016, accessed 20 December 2016).

[4] Mathers C Loncar D Projections of global mortality and burden of disease from 2002 to 2030. PLoS Med 2016; 3: e442. 10.1371/journal.pmed.0030442 PMC1664601 17132052

[5] Mannino DM Buist AS Global burden of COPD: risk factors, prevalence, and future trends. Lancet 2007; 370: 765–773. 17765526 10.1016/S0140-6736(07)61380-4

[6] National Institute for Health and Clinical Excellence (NICE). Chronic obstructive pulmonary disease: Costing report. https://www.nice.org.uk/guidance/cg101/resources/costing-report-134511805 (2011, accessed 11 January 2017).

[7] Graham V Lasserson T Rowe BH Antibiotics for acute asthma. Cochrane Database Syst Rev 2001; (3):CD002741. 10.1002/14651858.CD002741 11687022

[8] Wedzicha JA Seemungal TA COPD exacerbations: defining their cause and prevention. Lancet 2007; 370: 786–796. 17765528 10.1016/S0140-6736(07)61382-8 PMC7134993

[9] Vollenweider DJ Jarrett H Steurer-Stey CA Antibiotics for exacerbations of chronic obstructive pulmonary disease. Cochrane database Syst Rev 2012; 12: CD010257. 23235687 10.1002/14651858.CD010257

[10] Johnston SL Szigeti M pd Cross M Azithromycin for acute exacerbations of asthma: the AZALEA randomized clinical trial. JAMA Intern Med 2016; 176: 1630–1637. 27653939 10.1001/jamainternmed.2016.5664

[11] British Thoracic Society. BTS/SIGN British guideline on the management of asthma. https://www.brit-thoracic.org.uk/standards-of-care/guidelines/btssign-british-guideline-on-the-management-of-asthma/ (2016, accessed 11 January 2017).

[12] Vanderweil SG Tsai CL Pelletier AJ Inappropriate use of antibiotics for acute asthma in United States emergency departments. Acad Emerg Med 2008; 15: 736–43. 18627585 10.1111/j.1553-2712.2008.00167.x

[13] WHO. Antimicrobial Resistance. Global Report on Surveillance. http://www.who.int/drugresistance/en/ (2014, accessed 1 January 2017).

[14] World Health Organization. Global action plan on antimicrobial resistance. http://www.who.int/antimicrobial-resistance/publications/global-action-plan/en/ (2015, accessed 1 January 2017). 10.7196/samj.9644 26242647

[15] Laxminarayan R Heymann DL Challenges of drug resistance in the developing world. BMJ 2012; 344: e1567. 22491075 10.1136/bmj.e1567

[16] Forum of International Respiratory Societies. Respiratory diseases in the world Realities of Today – Opportunities for Tomorrow. Sheffield, UK https://www.ersnet.org/pdf/publications/firs-world-report.pdf (2013, accessed 2 March 2017).

[17] Center for Disease Dynamics E& P. ResistanceMap - Antibiotic Resistance. Antibiotic Resistance Map. https://resistancemap.cddep.org/AntibioticResistance.php (2015, accessed 2 March 2017).

[18] Falagas ME Avgeri SG Matthaiou DK Short- versus long-duration antimicrobial treatment for exacerbations of chronic bronchitis: a meta-analysis. J Antimicrob Chemother 2008; 62: 442–450. 18467303 10.1093/jac/dkn201

[19] Higgins J Green S (eds). The Cochrane Collaboration. Cochrane Handbook for Systematic Reviews of Interventions Version 5.1.0 [March 2011], www.handbook.cochrane.org (accessed 27 February 2016).

[20] The Nordic Cochrane Centre TCC. Review Manager (RevMan) [Computer program]. 2014; Version 5.3.

[21] Moussaoui R Roede BM Speelman P Short-course antibiotic treatment in acute exacerbations of chronic bronchitis and COPD: a meta-analysis of double-blind studies. Thorax 2008; 63: 415–422. 18234905 10.1136/thx.2007.090613

[22] Bennett JB Crook SJ Shaw EJ A randomized double blind controlled trial comparing two amoxycillin regimens in the treatment of acute exacerbations of chronic bronchitis. J Antimicrob Chemother 1988; 21: 225–232. 3283093 10.1093/jac/21.2.225

[23] Chodosh S Deabate CA Haverstock D Regular article: short-course moxifloxacin therapy for treatment of acute bacterial exacerbations of chronic bronchitis. Respir Med 2000; 94: 18–27. 10714475 10.1053/rmed.1999.0708

[24] Graham VL Knowles G Milton A Routine antibiotics in hospital management of acute asthma. Lancet 1982; 319: 418–421. 10.1016/s0140-6736(82)91619-1 6121090

[25] Johnston SL Blasi F Black PN The effect of telithromycin in acute exacerbations of asthma. N Engl J Med 2006; 354: 1589–1600. 16611950 10.1056/NEJMoa044080

[26] Gotfried M Notario G Spiller J Comparative efficacy of once daily, 5-day short-course therapy with clarithromycin extended-release versus twice daily, 7-day therapy with clarithromycin immediate-release in acute bacterial exacerbation of chronic bronchitis. Curr Med Res Opin 2005; 21: 245–254. 15801995 10.1185/030079905X26243

[27] Langan CE Zuck P Vogel F Randomized, double-blind study of short-course (5 day) grepafloxacin versus 10 day clarithromycin in patients with acute bacterial exacerbations of chronic bronchitis. J Antimicrob Chemother 1999; 44: 515. 10588313 10.1093/jac/44.4.515

[28] Lorenz J Steinfeld P Drath L Efficacy and tolerability of 5- vs 10-day cefixime therapy in acute exacerbations of chronic bronchitis. Clin Drug Investig 1998; 15: 13. 10.2165/00044011-199815010-00002 18370461

[29] Masterton RG Burley CJ Original article: randomized, double-blind study comparing 5- and 7-day regimens of oral levofloxacin in patients with acute exacerbation of chronic bronchitis. Int J Antimicrob Agents 2001; 18: 503–512. 11738336 10.1016/s0924-8579(01)00435-6

[30] Roede BM Bresser P El R Three vs. 10 days of amoxycillin-clavulanic acid for type 1 acute exacerbations of chronic obstructive pulmonary disease: a randomised, double-blind study. Clin Microbiol Infect 2007; 13: 284–290. 17391383 10.1111/j.1469-0691.2006.01638.x

[31] Sethi S Breton J Wynne B Efficacy and safety of pharmacokinetically enhanced amoxicillin-clavulanate at 2,000/125 milligrams twice daily for 5 days versus amoxicillin-clavulanate at 875/125 milligrams twice daily for 7 days in the treatment of acute exacerbations of chronic. Antimicrob Agents Chemother 2005; 49: 153–160. 15616290 10.1128/AAC.49.1.153-160.2005 PMC538920

[32] Pauwels R Global initiative for chronic obstructive lung diseases (GOLD): time to act. Eur Respir J 2001; 18(6): 901–902. 11829093

[33] National Institute for Health and Care Excellence (NICE). Chronic obstructive pulmonary disease in over 16s: diagnosis and management. Guidance and guidelines. https://www.nice.org.uk/guidance/cg101 (2010, accessed 1 January 2017). 31211541

[34] Dimopoulos G Matthaiou DK Karageorgopoulos DE Short- versus long-course antibacterial therapy for community-acquired pneumonia: a meta-analysis. Drugs 2008; 68: 1841–1854. 18729535 10.2165/00003495-200868130-00004

[35] Altamimi S Khalil A Khalaiwi KA Short-term late-generation antibiotics versus longer term penicillin for acute streptococcal pharyngitis in children In: Altamimi S (ed) Cochrane Database of Systematic Reviews 2012; 8: CD004872. DOI: 10.1002/14651858.CD004872.pub3. 10.1002/14651858.CD004872.pub3 PMC11984625 22895944

[36] Haider BA Lassi ZS Bhutta ZA Short-course versus long-course antibiotic therapy for non-severe community-acquired pneumonia in children aged 2 months to 59 months In: Bhutta ZA (ed.) Cochrane Database of Systematic Reviews. 2008; 2: CD005976. DOI: 10.1002/14651858.CD005976.pub2. 10.1002/14651858.CD005976.pub2 18425930

[37] Kallstrom TJ Evidence-based asthma management. Respir Care 2004; 49: 783–792. 15222910

