# Supplementary material for: Does antibiotic treatment duration affect the outcomes of exacerbations of asthma and COPD? A systematic review
Source: Chron Respir Dis. 2017 Dec 12;15(3):225–40. doi: 10.1177/1479972317745734 (PMC6100164; doi:10.1177/1479972317745734)
Supplement: Supplemental Material, Review_COPD_Asthma_Abx_supplementary_data_1.0_-_revised_1.0 - Does antibiotic treatment duration affect the outcomes of exacerbations of asthma and COPD? A systematic review [file Review_COPD_Asthma_Abx_supplementary_data_1.0_-_revised_1.0.pdf]

Does antibiotic treatment duration affect the outcomes of exacerbations of asthma and COPD? A Systematic Review

Supplementary data

Study or Subgroup	<6 days treatment Events	<6 days treatment Total	≥7 days treatment Events	≥7 days treatment Total	Weight	Risk Ratio M-H, Fixed, 95% CI
Bennett 1988	0	20	2	21	2.9%	0.21 [0.01, 4.11]
Chodosh 2000	12	312	23	302	27.4%	0.51 [0.26, 1.00]
DeAbate 1999	17	195	13	193	15.3%	1.29 [0.65, 2.59]
Gotfried 2001	9	174	11	175	12.8%	0.82 [0.35, 1.94]
Gotfried 2005	9	240	10	245	11.6%	0.92 [0.38, 2.22]
Langan 1999	5	273	12	268	14.2%	0.41 [0.15, 1.15]
Lorenz 1998	0	110	3	111	4.1%	0.14 [0.01, 2.76]
Masterton 2001	8	268	10	262	11.8%	0.78 [0.31, 1.95]
**Total (95% CI)**		**1592**		**1577**	**100.0%**	**0.71 [0.52, 0.98]**
Total events	60		84			

Heterogeneity: Chi² = 7.19, df = 7 (P = 0.41); I² = 3%
Test for overall effect: Z = 2.09 (P = 0.04)

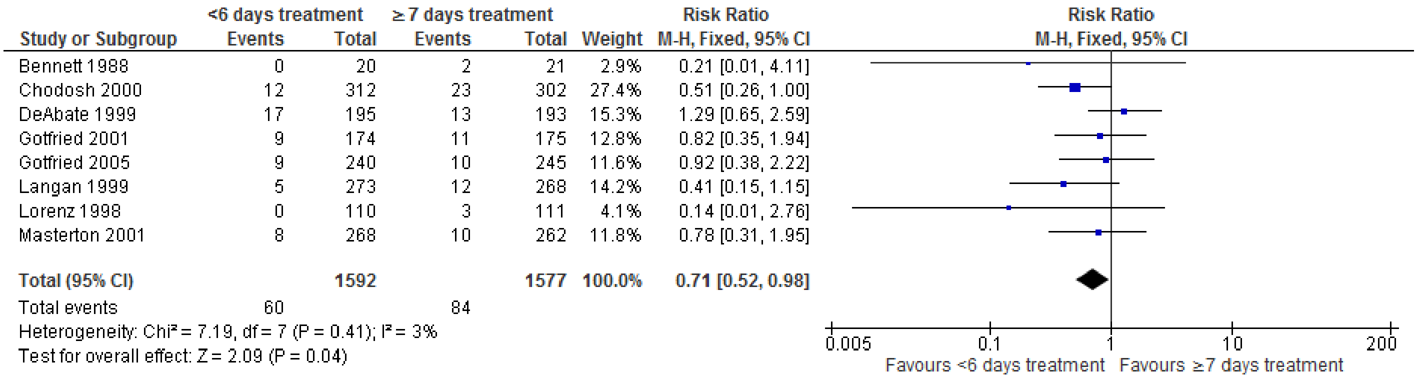

Supplementary Figure 1 – Forest plot of nausea (adverse outcome), < 6 versus ≥ 7 days antibiotic duration

Study or Subgroup	<6 days treatment Events	<6 days treatment Total	≥7 days treatment Events	≥7 days treatment Total	Weight	Risk Ratio M-H, Fixed, 95% CI
Chodosh 2000	15	312	18	302	13.9%	0.81 [0.41, 1.57]
DeAbate 1999	6	195	6	193	4.6%	0.99 [0.32, 3.02]
Gotfried 2001	12	174	11	175	8.3%	1.10 [0.50, 2.42]
Langan 1999	3	273	5	268	3.8%	0.59 [0.14, 2.44]
Lorenz 1998	13	110	20	111	15.1%	0.66 [0.34, 1.25]
Masterton 2001	13	268	7	262	5.4%	1.82 [0.74, 4.48]
Sethi 2005	74	443	65	450	48.9%	1.16 [0.85, 1.57]
**Total (95% CI)**		**1775**		**1761**	**100.0%**	**1.03 [0.82, 1.29]**
Total events	136		132			

Heterogeneity: Chi² = 5.07, df = 6 (P = 0.53); I² = 0%
Test for overall effect: Z = 0.29 (P = 0.77)

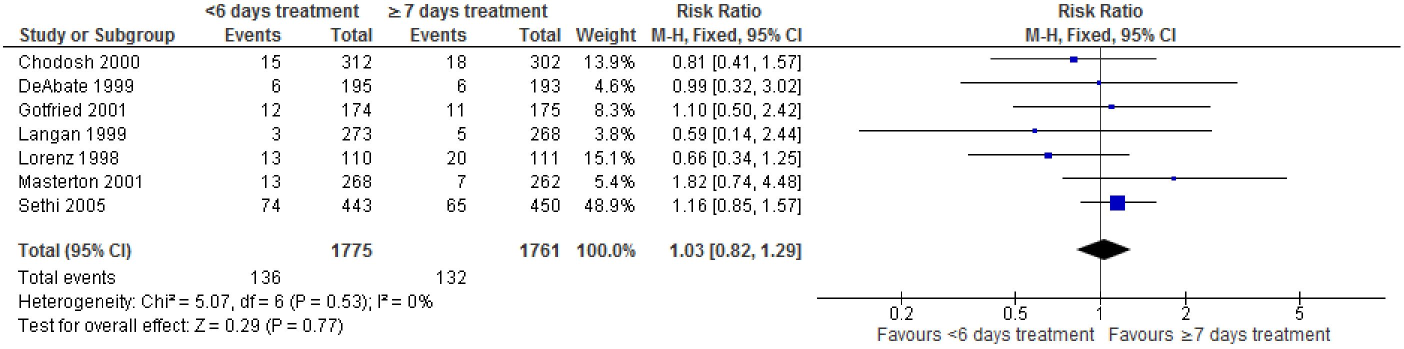

Supplementary Figure 2 – Forest plot of diarrhea (adverse outcome), < 6 versus ≥ 7 days antibiotic duration

Study or Subgroup	< 6 days treatment Events	Total	≥ 7 days treatment Events	Total	Weight	Risk Ratio (Non-event) M-H, Fixed, 95% CI
Chodosh 2000	127	135	138	145	19.3%	1.23 [0.46, 3.29]
Lorenz 1998	25	35	14	17	11.6%	1.62 [0.51, 5.13]
Sethi 2005	95	118	93	117	69.1%	0.95 [0.57, 1.58]
**Total (95% CI)**		**288**		**279**	**100.0%**	**1.08 [0.71, 1.65]**
Total events	247		245			

Heterogeneity: Chi² = 0.78, df = 2 (P = 0.68); I² = 0%
Test for overall effect: Z = 0.36 (P = 0.72)

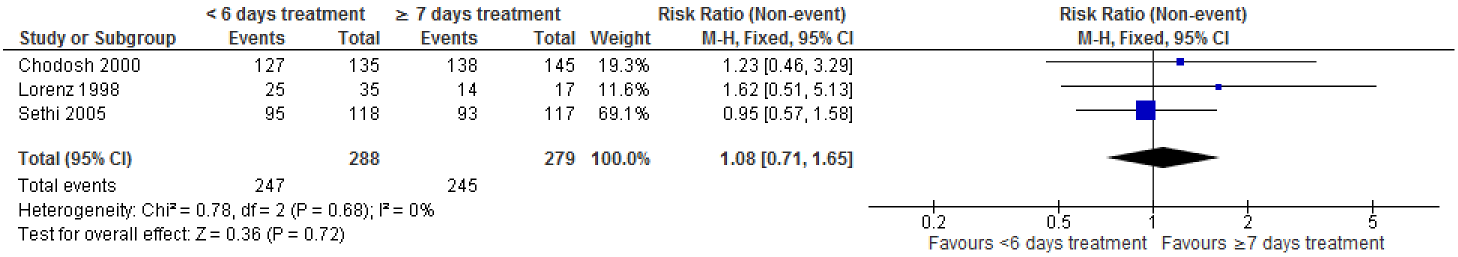

Supplementary Figure 3 – Eradication or presumed eradication of bacteria from sputum within 6 days of treatment completion, < 6 versus ≥ 7 days antibiotic duration

Study or Subgroup	< 6 days treatment Events	Total	≥ 7 days treatment Events	Total	Weight	Risk Ratio (Non-event) M-H, Fixed, 95% CI
Chodosh 2000	127	143	135	148	14.1%	1.27 [0.64, 2.55]
DeAbate 1999	104	138	94	123	33.9%	1.04 [0.68, 1.61]
Gotfried 2001	85	87	75	80	5.8%	0.37 [0.07, 1.84]
Gotfried 2005 (1)	82	94	91	102	11.7%	1.18 [0.55, 2.55]
Lorenz 1998	24	35	12	17	7.5%	1.07 [0.44, 2.59]
Masterton 2001	85	112	80	101	24.4%	1.16 [0.70, 1.92]
Roede 2007	9	11	6	8	2.6%	0.73 [0.13, 4.13]
**Total (95% CI)**		**620**		**579**	**100.0%**	**1.08 [0.83, 1.39]**
Total events	516		493			

Heterogeneity: Chi² = 2.29, df = 6 (P = 0.89); I² = 0%
Test for overall effect: Z = 0.56 (P = 0.58)

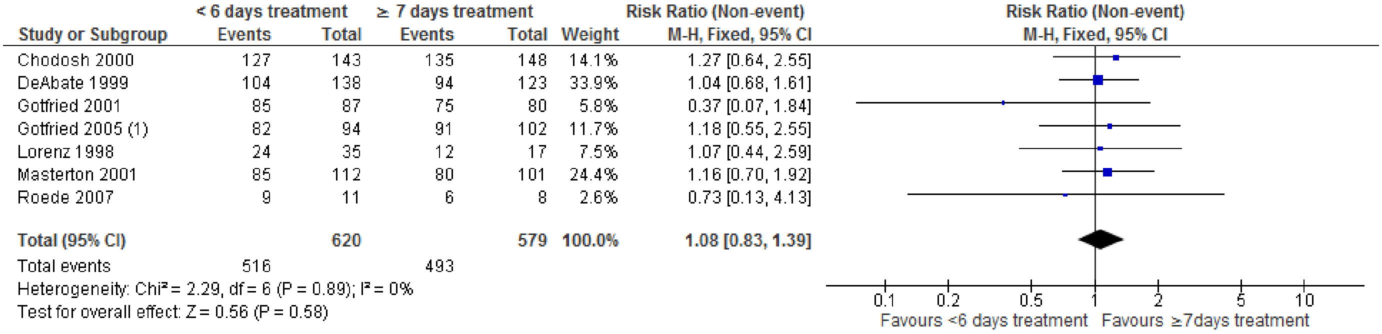

Footnotes
(1) Eradication only

Supplementary Figure 4 - Eradication or presumed eradication of bacteria from sputum within 7-23 days of treatment completion, < 6 versus ≥ 7 days antibiotic duration

Study or Subgroup	< 6 days treatment Events	Total	≥ 7 days treatment Events	Total	Weight	Risk Ratio M-H, Fixed, 95% CI
Chodosh 2000	274	288	266	281	27.8%	1.01 [0.97, 1.04]
Langan 1999	141	273	149	268	0.0%	0.93 [0.79, 1.09]
Lorenz 1998	77	108	73	109	7.5%	1.06 [0.89, 1.27]
Masterton 2001	214	268	212	262	22.1%	0.99 [0.91, 1.07]
Sethi 2005	414	443	417	450	42.6%	1.01 [0.97, 1.05]
**Total (95% CI)**		**1107**		**1102**	**100.0%**	**1.01 [0.98, 1.04]**
Total events	979		968			

Heterogeneity: Chi² = 0.61, df = 3 (P = 0.89); I² = 0%
Test for overall effect: Z = 0.46 (P = 0.65)

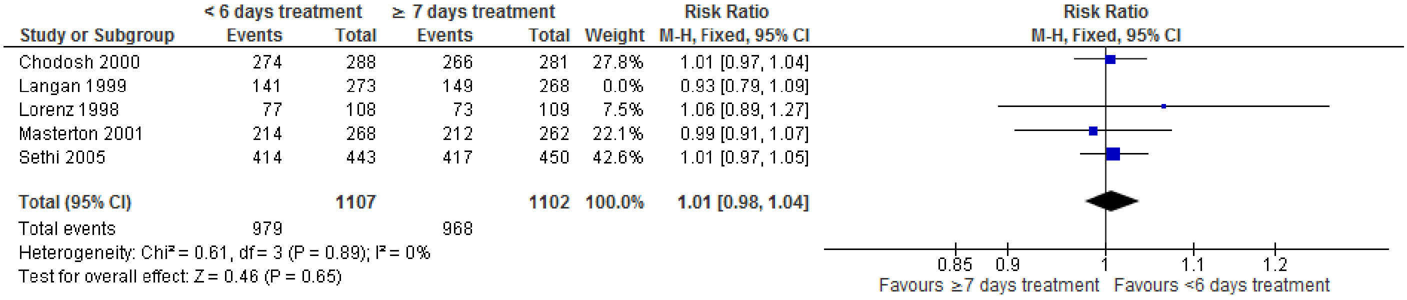

Supplementary Figure 5 - Forest plot of clinical success within 6 days of treatment completion, < 6 versus ≥ 7 days antibiotic duration, sensitivity analysis excluding Langan et al.

Study or Subgroup	< 6 days treatment Events	Total	≥ 7 days treatment Events	Total	Weight	Risk Ratio M-H, Fixed, 95% CI
DeAbate 1999	128	195	122	193	15.1%	1.04 [0.90, 1.20]
Gotfried 2001	134	174	135	175	16.5%	1.00 [0.89, 1.12]
Langan 1999	113	273	127	268	0.0%	0.87 [0.72, 1.06]
Masterton 2001	174	268	177	262	22.0%	0.96 [0.85, 1.09]
Roede 2007	13	23	14	25	1.6%	1.01 [0.61, 1.66]
Sethi 2005	379	443	368	450	44.8%	1.05 [0.99, 1.11]
**Total (95% CI)**		**1103**		**1105**	**100.0%**	**1.02 [0.97, 1.07]**
Total events	828		816			

Heterogeneity: Chi² = 1.90, df = 4 (P = 0.75); I² = 0%
Test for overall effect: Z = 0.72 (P = 0.47)

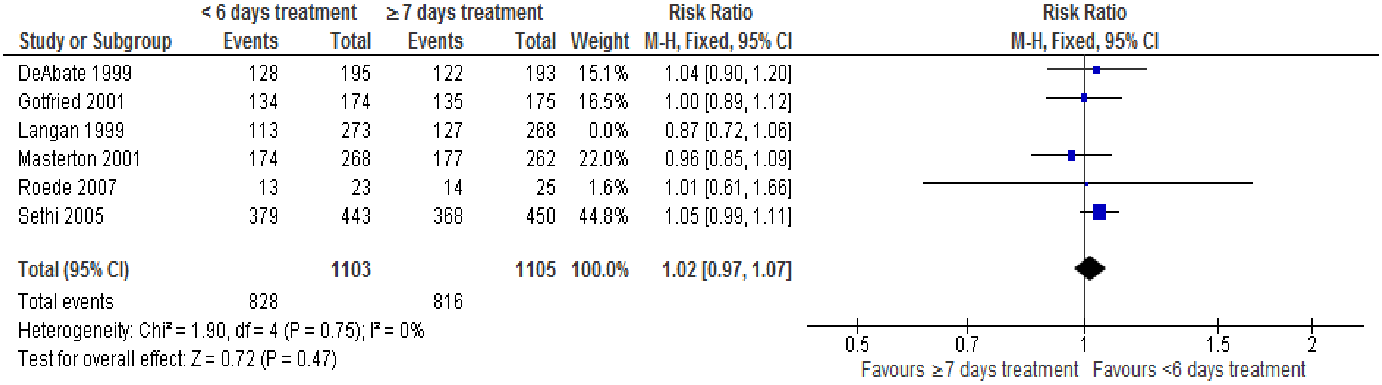

Supplementary Figure 6 - Forest plot of clinical success > 20 days after treatment completion, < 6 versus ≥ 7 days antibiotic duration, sensitivity analysis excluding Langan et al.

Study or Subgroup	< 6 days treatment Events	Total	≥ 7 days treatment Events	Total	Weight	Risk Ratio M-H, Fixed, 95% CI
Bennett 1988	1	20	3	21	0.6%	0.35 [0.04, 3.09]
Chodosh 2000	81	312	91	302	19.4%	0.86 [0.67, 1.11]
DeAbate 1999	56	195	69	193	14.6%	0.80 [0.60, 1.07]
Gotfried 2005	31	240	45	245	9.4%	0.70 [0.46, 1.07]
Langan 1999	10	273	13	268	0.0%	0.76 [0.34, 1.69]
Lorenz 1998	15	110	21	111	4.4%	0.72 [0.39, 1.32]
Masterton 2001	52	268	51	262	10.8%	1.00 [0.70, 1.41]
Roede 2007	1	21	5	25	1.0%	0.24 [0.03, 1.88]
Sethi 2005	162	443	191	450	39.8%	0.86 [0.73, 1.01]
**Total (95% CI)**		**1609**		**1609**	**100.0%**	**0.84 [0.75, 0.94]**
Total events	399		476			

Heterogeneity: Chi² = 4.14, df = 7 (P = 0.76); I² = 0%
Test for overall effect: Z = 3.12 (P = 0.002)

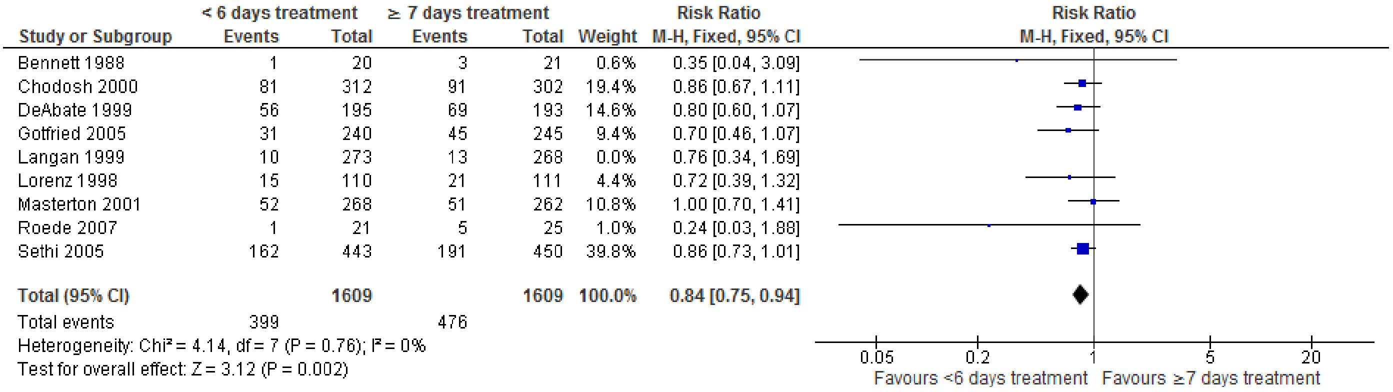

Supplementary Figure 7 - Forest plot of overall adverse events, < 6 versus ≥ 7 days antibiotic duration, sensitivity analysis excluding Langan et al.

Study or Subgroup	<6 days treatment Events	Total	≥7 days treatment Events	Total	Weight	Risk Ratio M-H, Fixed, 95% CI
Bennett 1988	0	20	2	21	3.3%	0.21 [0.01, 4.11]
Chodosh 2000	12	312	23	302	31.9%	0.51 [0.26, 1.00]
DeAbate 1999	17	195	13	193	17.8%	1.29 [0.65, 2.59]
Gotfried 2001	9	174	11	175	15.0%	0.82 [0.35, 1.94]
Gotfried 2005	9	240	10	245	13.5%	0.92 [0.38, 2.22]
Langan 1999	5	273	12	268	0.0%	0.41 [0.15, 1.15]
Lorenz 1998	0	110	3	111	4.8%	0.14 [0.01, 2.76]
Masterton 2001	8	268	10	262	13.8%	0.78 [0.31, 1.95]
**Total (95% CI)**		**1319**		**1309**	**100.0%**	**0.76 [0.54, 1.07]**
Total events	55		72			

Heterogeneity: Chi² = 5.80, df = 6 (P = 0.45); I² = 0%
Test for overall effect: Z = 1.59 (P = 0.11)

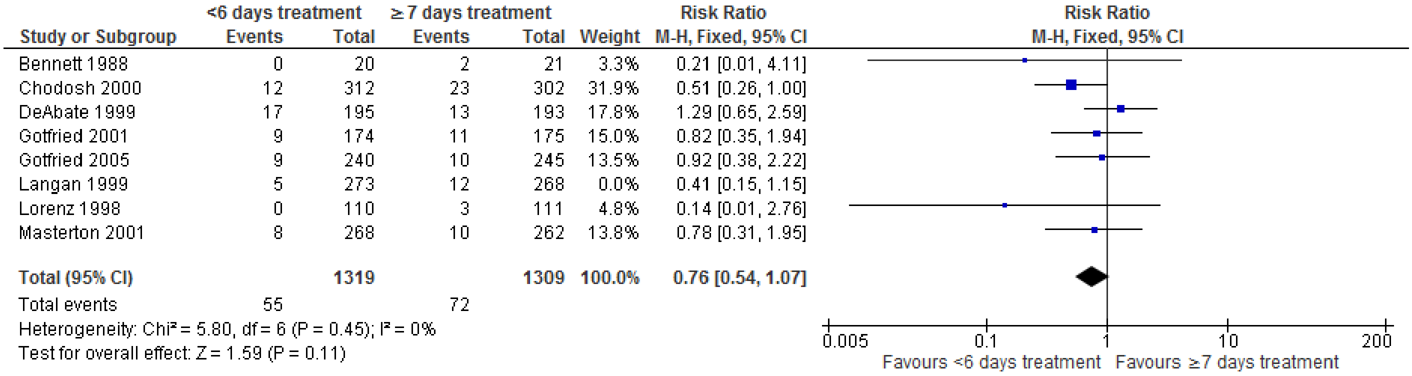

Supplementary Figure 8 - Forest plot of nausea (adverse event), < 6 versus ≥ 7 days antibiotic duration, sensitivity analysis excluding Langan et al.

Study or Subgroup	<6 days treatment Events	<6 days treatment Total	≥7 days treatment Events	≥7 days treatment Total	Weight	Risk Ratio M-H, Fixed, 95% CI
Chodosh 2000	15	312	18	302	14.4%	0.81 [0.41, 1.57]
DeAbate 1999	6	195	6	193	4.8%	0.99 [0.32, 3.02]
Gotfried 2001	12	174	11	175	8.7%	1.10 [0.50, 2.42]
Langan 1999	3	273	5	268	0.0%	0.59 [0.14, 2.44]
Lorenz 1998	13	110	20	111	15.7%	0.66 [0.34, 1.25]
Masterton 2001	13	268	7	262	5.6%	1.82 [0.74, 4.48]
Sethi 2005	74	443	65	450	50.9%	1.16 [0.85, 1.57]
**Total (95% CI)**		**1502**		**1493**	**100.0%**	**1.05 [0.84, 1.32]**
Total events	133		127			

Heterogeneity: Chi² = 4.45, df = 5 (P = 0.49); I² = 0%
Test for overall effect: Z = 0.43 (P = 0.67)

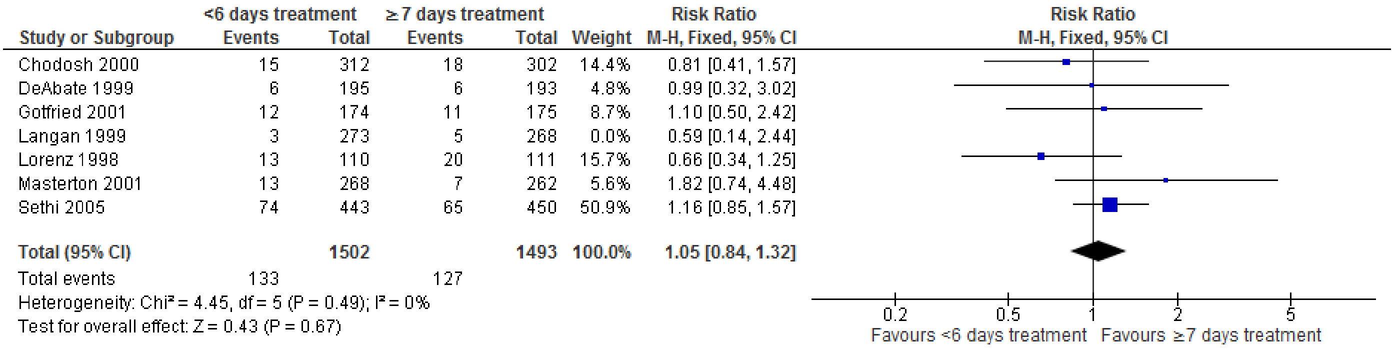

Supplementary Figure 9 - Forest plot of diarrhea (adverse event), < 6 versus ≥ 7 days antibiotic duration, sensitivity analysis excluding Langan et al.

Supplementary Table 1 - Overview of clinical outcome assessment by study

Study	Early follow up	Medium / Late follow up	Outcomes
Bennett 1988 [28]	0 days after treatment completion	12 months	Absence of mucoid sputum<br>At 12 months: number of exacerbations; time to first exacerbation
Chodosh 2000 [29]	0-6 days after treatment completion	7-17 days after treatment completion	Clinical success: resolution of acute signs / symptoms related to infection or sufficient improvement so that additional / alternative antibiotics were not required
DeAbate 1999 [36]		21-24 days after treatment completion	Clinical cure or improvement
Gotfried 2001 [37]		7-14 days after treatment completion<br>Extended follow up (21-28 days after treatment completion)	Clinical cure: resolution of all signs & symptoms of acute exacerbation of chronic bronchitis which were present at study entry, without need for further antibiotics
Gotfried 2005 [25]		Days 10-14 after treatment completion	Clinical cure: resolution of signs / symptoms of acute exacerbation of chronic bronchitis
Langan 1999 [27]	1-3 days after treatment completion	21-28 days after treatment completion	Satisfactory clinical response: cure or signs / symptoms of acute exacerbation of chronic bronchitis have improved / resolved at post-

			treatment visit (day 1-3) and absent at follow up visit or incomplete resolution at follow up visit
Lorenz 1998 [30]	0-3 days after treatment completion	17-23 days after treatment completion	Clinical success: cure or improvement of acute bacterial exacerbation based on volume and nature of sputum and symptoms of breathlessness
Masterton 2001[23]	1-3 days after treatment completion	7-10 days after treatment completion (primary outcome)<br>4-5 weeks after treatment completion	Clinical success: resolution of all infection related signs / symptoms or return to pre-infection state or improvement with no subsequent antibacterial treatment indicated
Roede 2007 [26]		11 days after treatment completion<br>3 months (minus 10 days) after treatment completion	Cure: resolution of acute signs / symptoms of acute exacerbation of chronic bronchitis back to baseline
Sethi 2005 [24]	2-4 days after treatment completion	7-14 days after treatment completion (primary outcome)<br>21-28 days after treatment completion	Clinical success: sufficient improvement or resolution of the signs and symptoms of acute exacerbation of chronic bronchitis recorded at the screening such that no additional antibacterial therapy was prescribed for the episode of acute exacerbation of chronic bronchitis
